# The crystallization behavior of the aqueous solution of CaCl_2_ salt in a drop and a layer

**DOI:** 10.1038/s41598-019-57169-1

**Published:** 2020-01-14

**Authors:** S. Y. Misyura

**Affiliations:** Kutateladze Institute of Thermophysics Siberian Branch, Russian Academy of Sciences, 1 Lavrentyev Ave., Novosibirsk, 630090 Russia

**Keywords:** Chemical engineering, Chemical engineering, Mechanical engineering, Mechanical engineering

## Abstract

Non-isothermal evaporation during crystallization of CaCl_2_ salt in a droplet and a thin layer on a hot wall has been investigated experimentally. The growth of salt crystal hydrates (CaCl_2_·2H_2_O) on the interface has been studied. It has been found that the change in the initial salt concentration leads to different crystallization rates. The crystallization rate on the droplet interface is many times lower than for a thin layer. The description of crystallization in the salt solution droplet should take into account the crystallization anisotropy, which is associated with the direction of crystallization. The crystallization rate along the contact line of the droplet is many times higher than in the direction of the droplet radius. For a long time of crystallization, the area of the crystal film (outside the drop) increases several times. Four characteristic modes of crystallization to a drop of salt solution have been distinguished. When modeling crystallization, it is necessary to take into account multiple changes in the growth rate of salt crystallohydrates over time, as well as the anisotropic nature of crystallization.

## Introduction

In industrial technologies, several processes are often implemented simultaneously during crystallization: dissolution, dilution, heat transfer and evaporation. At that, liquid temperature, salt concentration and supersaturation change over time. Changes in boundary conditions over time significantly complicate the simulation. Spatial limitations change the heat transfer coefficient and crystallization rate. For the correct physical description of the complex non-stationary and non-isothermal processes, it is essential to review the literature in several related fields.

Non-isothermal evaporation of drops and films is also used in a wide range of technical problems: the fuel multicomponent systems with gas-droplet flows; the absorption heat pumps in power engineering^1,2^; chemical technologies. When modeling high-temperature evaporation of droplets and films, the physical and chemical properties of the wall have to be taken into account. The presence of water droplets and water vapor in the multicomponent fuel leads to lower combustion temperature and reduces harmful emissions during combustion^3^.

Modern technologies widely apply textured surfaces to control wettability and to intensify droplet and film evaporation^4–7^. The droplet evaporation rate may be effectively controlled by applying surfactants^8,9^. At that the evaporation rate becomes nonlinearly dependent on time. Characteristic modes of droplet evaporation with surfactant are considered in^9^.

Aqueous salt solutions are widely used in modern energy technologies: membrane technologies^10^ and ion transport^11,12^. Evaporation of salt solutions differs from pure water. Since the salt concentration increases with time, the evaporation rate, on the contrary, can fall many times, and the evaporation kinetics depends on the salt type^13,14^. The rapid increase in the salt concentration during boiling leads to the degeneration of the nucleate boiling and to the implementation of four evaporation modes, successively replacing each other^15,16^. Changes in the physical properties of aqueous solutions of salts with time of evaporation, significantly complicate the modeling of solutions. The properties of the aqueous solution of CaCl_2_ salt and equilibrium diagrams were studied in^17^.

Regularities of crystallization of salt solutions are important for describing numerous technologies associated with the creation of new materials, nanofilms with high heat and electrical conductivity, having properties of semiconductors and high strength. The properties of materials change when the crystallization rate and crystal forms vary^18–21^. The regularities of crystallization in salt solution drops and in double solutions are presented in^22–24^. It is characteristic that over evaporation time the crystal forms on the free surface of the droplet can vary and depend on the wettability. The beginning of crystallization in the drop is realized in the precursor film of the salt solution^25^, where the maximum supersaturation occurs. Collective effects and spontaneous crystallization in water droplets on the surface of heavy oil are considered in^26^. The main points of the theory of crystallization of salt solutions and the driving forces of crystallization are considered in^27^.

When describing evaporation and crystallization, it is necessary to estimate not only the rate of heat transfer in the liquid, but also convection in the gas phase^28–30^, which significantly increases the evaporation rate. The role of free convection in salt solution drops decreases with increasing evaporation time, since the salt concentration increases^31–33^. In this case, the heat transfer coefficient may have an extremum when approaching the crystallization. The Marangoni flow on the surface of a water drop at room temperature is suppressed by micron particles of dirt, which may be a surfactant^33^. With increasing wall temperature and heat flux, the thermal and the solutal Marangoni forces lead to intensive circulation of salt solution inside the droplet^31^.

The analysis of the literature has shown that the non-isothermal crystallization at high heat fluxes is influenced by many key factors, and this impact changes with time. At that, the heat transfer coefficient cannot be taken constant, as it is done in most approximate theoretical works. Factors that lead to a higher metastable area have not been thoroughly investigated. The objectives of these studies are to determine the effect of the initial salt concentration on the salt solution supersaturation and the crystallization rate, to compare the crystallization rate in different directions of the droplet, to compare the crystallization kinetics for a droplet and layer salt solution.

## Experimental Method

Experiments were carried out for a droplet and a thin layer of an aqueous solution of CaCl_2_ salt. The drop and layer evaporated on a horizontal metal hot wall. The wall roughness and the static contact angle *θ*_0_ of the droplet remained for a long time (several hours). When a sessile drop evaporates, various regimes are available for the behavior of the droplet contact line. For example, the constant contact radius (CCR) mode or the constant contact angle (CCA) mode (the CCR mode has *R* = const and *θ*_0_ = var., *R* is a droplet radius; the CCA mode has *θ*_0_ = const and *R* = var., *θ*_0_ is the droplet static contact angle). For all drops of salt solution (the wall has good wettability in the presented experiments), there was the CCR mode that was implemented before the crystallization began (during crystallization, of course, the CCR mode was maintained). Wettability affects induction time and crystallization rate. A significant increase in wettability will decrease free convection at the edges of the droplet, and will change the adhesion of impurity particles and microcrystalline nuclei relative to the solid wall. In this case, the induction period and the crystallization rate vary (since the supersaturation of solution will be higher). A change in wettability is not included in the purpose of this research. The change in wettability preserves the qualitative nature of the findings.

The duration of each experiment did not exceed 30 minutes. The droplet contact angle was measured before and after the experiment. Before measuring *θ*_0,_ the salt solution was removed, and the wall was repeatedly cleaned with water and alcohol. The initial values of the droplet contact angle for the CaCl_2_ aqueous salt solution (with the initial mass concentration of salt *C*_*m*0_ = 10%) varied within *θ*_0_ = 51–54°. For *C*_*m*0_ = 50%, the contact angle varied in the range 60–66°. Changes in the wall roughness (standard deviation) were within 1–2 µm for all experiments. All experiments were carried out at a constant wall temperature *T*_*w*_ = 75 °C, which was maintained quasi-constant (with an accuracy of 0.6–0.7 °C) in the automatic mode by adjusting the power of the electric heater. The heater with tungsten wire was located inside the metal working cylinder. The surface temperature *T*_*w*_ was measured by a thermocouple. The total error of *T*_*w*_ measurement did not exceed 1–1.2 °C. The temperature *T*_*s*_ of the liquid interface was measured by a thermal imager NEC-San Instruments (640 × 512 pixels and the resolution of 10 µm) with a maximum error of 1.5 °C. A detailed scheme of the setup are given in^14^.

The evaporation of aqueous salt solutions was realized at a relative air humidity of 34–40%. The temperature of the external air varied within 22–23 °C. The external air pressure was 1 bar. We used a particularly pure salt and distillate, which underwent a long degassing. The initial salt concentration *C*_*m*0_ was measured using densimeters. Droplet was applied to the wall surface using automatic densimeter (the electronic dispenser of Finnpipette Novus with an error of 0.5–0.7%) with a given rate of liquid run out and a given droplet volume. The droplet application process provided good repeatability of the droplet geometry. The diameter and height of the droplet changed by no more than 5–7%. Since the water from the liquid solution continuously evaporated, the current mass concentration of salt *C*_*m*_ increased over time. Values of *C*_*m*_ were measured using the gravimetric method. Changes in the liquid mass were recorded by scales, and the current salt concentration was determined with a maximum error of less than 8–10%. For all experiments, the initial droplet and the layer height were constant (*h* = 2.8 ± 0.1 mm).

Since the crystallization rate *j*_*cr*_ in the salt solution depends on the heat transfer, the heat transfer coefficients *α*_*s*_ were measured in the experiments. During crystallization, *α*_*s*_ changed slightly. In the calculations, the average values of *α*_*s*_ were used for the time of crystallization. The heat transfer coefficient *α*_*s*_ of the aqueous salt solution was determined experimentally on the basis of heat balance (Eq. 1). The thermal energy coming to the free liquid surface (*α*_*s*_(*T*_*w*_ − *T*_*s*_)) due to heat exchange in the liquid was spent on cooling from water evaporation (*r*_*e*_*j*_*e*_) and from salt dilution (*q*_*s*_), as well as on cooling from convection in gas (*α*_*g*_(*T*_*s*_ − *T*_0_)) and radiation (*q*_*r*_),

1 $${\alpha }_{s}({T}_{w}-{T}_{s})+({r}_{cr}{j}_{cr})/F\approx {\alpha }_{s}({T}_{w}-{T}_{s})=({r}_{e}{j}_{e})/F+{\alpha }_{g}({T}_{s}-{T}_{0})+{q}_{s}+{q}_{r}$$

where *r*_*e*_ is the latent evaporation heat, *r*_*cr*_ is the latent crystallization heat, *j*_*e*_ is the evaporation rate of the salt solution, *j*_*cr*_ is the crystallization rate, *α*_*g*_ is the heat transfer coefficient of the gas (air + water vapor), *T*_0_ is the air temperature, *q*_*r*_ is the radiation heat, and *q*_*s*_ is the dilution heat, and *F* is the surface area. Since the thickness of the crystalline crust was about 0.02 mm, and the temperature increase due to crystallization was less than 1 °C^20^, the value of the heat of crystallization will be of the second order of smallness compared to the evaporation heat. Then Eq. 1 can be simplified and *α*_*s*_ may be defined according to Eq. 2,

2 $${\alpha }_{s}=(({r}_{e}{j}_{e})/F+{\alpha }_{g}({T}_{s}-{T}_{0})+{q}_{s}+{q}_{r})/({T}_{w}-{T}_{s})$$

where *j*_*e*_ is determined experimentally by the gravimetric method. The temperature difference *T*_*w*_ − *T*_*s*_ is also determined experimentally. A detailed description of the method of determining *α*_*s*_ is presented in^13,14^. The maximum relative measurement error of *α*_*s*_ was less than 18% and corresponded to the minimum evaporation rate.

To assess the convection effect on the crystallization (*j*_*cr*_), the average velocity (*U*) in the droplet horizontal section was measured using the Particle Image Velocity (PIV) method (the dual Nd – YAG laser Quantel EverGreen 70; the wavelength of 532 nm; the repetition rate of 4 Hz, the pulse energy of 70 mJ; the camera ImperX IGV-B2020M with the image resolution of 2048 × 2048 pix, frequency of shooting of 4 fps, bit width of 8 bit, and macro lens Nikon 200 mm f/4 AF-D Macro). Measurements of velocity fields were realized in a horizontal section of the drop at a distance of 0.6 mm from the wall surface. TiO_2_ particles were used to visualize the velocity field. The particles size was less than 1 μm. A detailed PIV method for the velocity measuring in a droplet of aqueous salt solution was presented in^31,32^.

## Experimental Data and Analysis

For the description of crystallization of one-component fluid, one can use expression (3)

3 $${j}_{cr}=k\Delta {T}_{cr}$$

**Figure 1:**
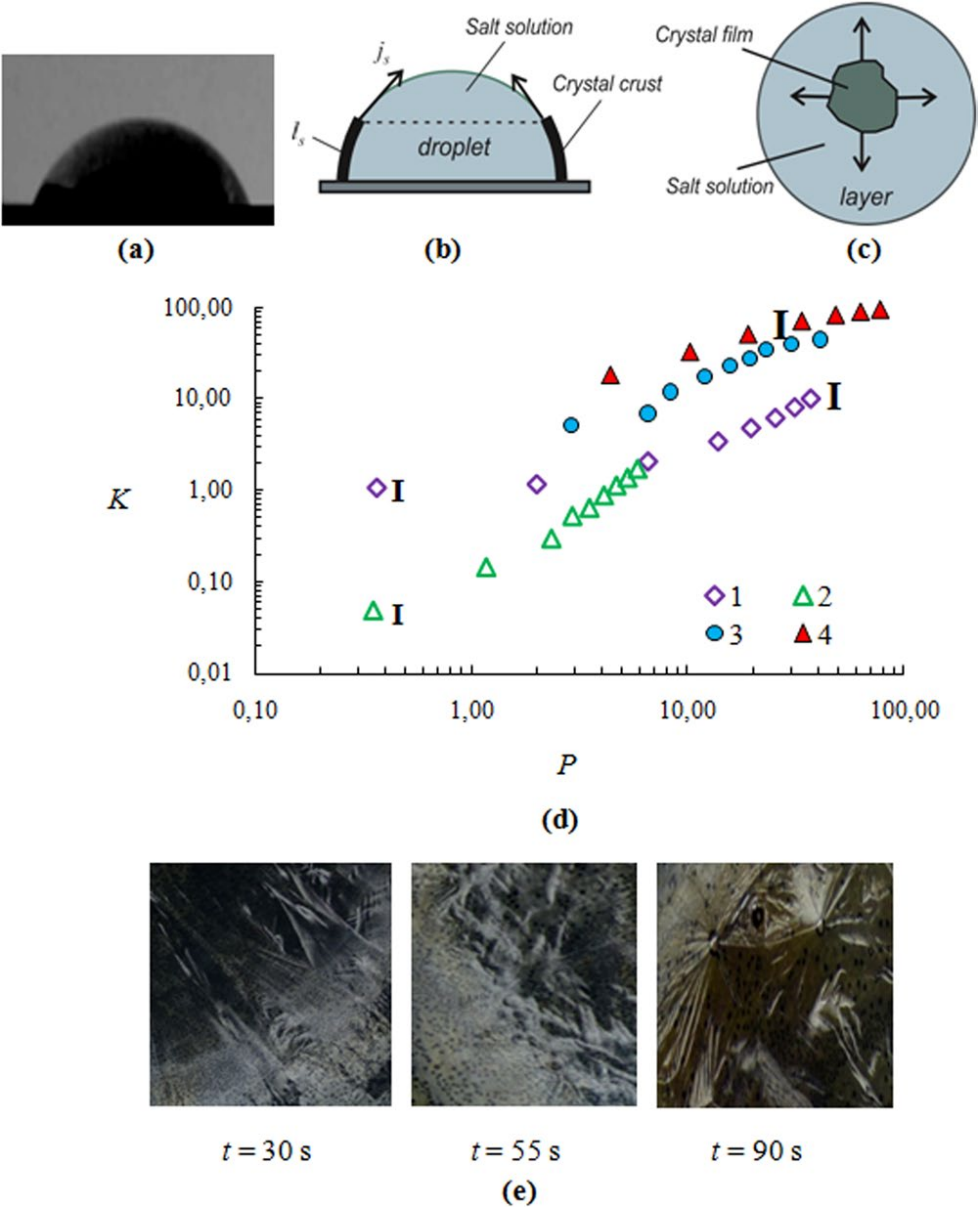
(**a**) Photo of the droplet (*d*_0_ = 6 mm); (**b**) crystal film motion on the droplet surface; (**c**) crystal front direction on the free liquid surface for a thin layer; (**d**) dependence of the dimensionless coordinate for the CaCl_2_ salt solution *K* (*K* = *fl*_*s*_) on *P* (*P* = *af*  ^2^*t*) (the initial drop height *h*_0_ = 2.8 mm; *T*_*w*_ = 75 °C; Curves 1 and 2 for the drop; Curves 3 and 4 for the liquid layer): 1 – the initial salt concentration *C*_*m*0_ = 35%; 2 – *C*_*m*0_ = 50%; 3 – *C*_*m*0_ = 35%; 4 – *C*_*m*0_ = 15%; (**e)** Photos of crystal hydrates CaCl_2_·2H_2_O.

The crystallization rate *j*_*cr*_ is proportional to the temperature difference *∆T*_*cr*_ between the solution temperature at the crystallization front and the equilibrium temperature^34^. The crystallization kinetics of the solution binds the crystallization rate *j*_*cr*_ with the supersaturation *S* as follows (4)^27^,

4 $${j}_{cr} \sim exp(-16\pi {\sigma }^{3}{v}^{2}/(3{k}^{3}{T}^{3}{(lnS)}^{2}))$$

where *T* is the absolute solution temperature; *k* is the Boltzmann constant; *σ* is the interfacial tension of the solution; *v* is the molecular volume, and *S* is the supersaturation. *S* = *C*_*s*_/*C*_*cr*_, where *C*_*s*_ is the salt concentration in a supersaturated state of the salt solution and *C*_*cr*_ is the salt concentration for a saturated state of the aqueous salt solution. The principal difference between (4) and (3) is that the driving force of crystallization depends not only on the temperature, but also on the concentration of components. Supersaturation *S* is a function of temperature and concentration. To be more precise, the deviation from the saturation curve depends on the parameters *T* and *C*. The realization of spontaneous homogeneous crystallization requires extremely large deviations from the saturation curve. The present experiments consider the growth of the crystal film from one large crystal on the free surface of the droplet and layer. In this case, heterogeneous crystallization is realized. There are maximum three or four growing crystals along the contact line, rather than tens or hundreds of crystals. At the same time, during crystallization, the salt concentration and supersaturation change, but new crystal centers do not appear. In connection with the above, expression (4) can be used only for qualitative evaluation, which relates the crystal growth rate to supersaturation. In view of the above, a key role in the crystal growth is played not only by *S*, but the heat exchange. Curves in Fig. 1 are given in dimensionless coordinates taking into account the calculated values of the heat transfer coefficients^34^. This generalization allows taking into account the role of heat transfer and the role of supersaturation is more clearly visible, which explains the divergence of curves.

Table 1 shows the calculated values of *α*_*s*_ in accordance with expression (2).

**Table 1:** The heat transfer coefficient *α*_*s*_ of the droplet of CaCl_2_ salt solution (*Cm*_0_ – the initial salt concentration).

	*Cm*_0_ = 15%,	*Cm*_0_ = 35%,	*Cm*_0_ = 50%,
*α*_*s*_, W/(Km^2^)	1314	710	190

With the growth of the initial salt concentration *α*_*s*_ decreases, since the viscosity of the salt solution increases many times. The neglect of *α*_*s*_ will result in incorrect interpretation of the results. The values of heat transfer coefficients in Table 1 differ many times (the maximum difference is almost 7 times).

Figure 1(a) shows the photo of the droplet profile. The crystal film moves in the direction from the contact line of the droplet to its center (Fig. 1(b)). For the layer, the crystals form near the center, as in this area there is the lowest temperature *T*_*s*_. The crystallization front for the layer has a direction from the center to the edge (Fig. 1(c)). As the film moves, the surface area of the film increases and the liquid decreases with time. Figure 1(d) represents experimental curves in dimensionless coordinates *K* and *P*^34^ (*K* = *fl*_*s*_, *P* = *af*^2^*t*, *f* = *α*_*s*_/*λ*_*s*_, *α*_*s*_ is the heat transfer coefficient of the aqueous salt solution, *a* is the thermal diffusivity of the salt solution, *t* is the time during crystallization, *λ*_*s*_ is the thermal conductivity of the CaCl_2_ salt solution, and *l*_*s*_ is the crystal front displacement on the free surface of a droplet or a thin layer (Fig. 1(b,c)). From Fig. 1(d) it follows that the curves for the droplet are substantially lower than for the layer. In addition, the crystalline front propagation depends on the initial salt concentration *C*_*m*0_. With the growth of *C*_*m*0_, the values of *K* decrease, i.e., the crystallization front rate (hereinafter, the crystallization rate) increases. Thus, if crystallization depended only on the thermophysical properties of the solution and the heat transfer coefficient, the curves 1–4 would coincide. The divergence of the curves is probably related to the dependence of crystallization on *S*. Supersaturation *S*, in turn, depends on the initial salt concentration and on the direction of crystallization (from the place of origin of the first rapidly growing crystal center). For a drop, the first crystal center grows in the vicinity of the contact line, and for a layer – near the center. The higher crystallization rate for the layer than for the droplet can be explained by different induction time (the time from the moment of saturation to the moment of the onset of the crystal growth, i.e. the duration of the metastable state). For the droplet, supersaturation was lower because of the smaller induction time. It is well known that in the presence of the surface flow of Marangoni, a high concentration of microscopic particles in the form of surfactants occurs at the drop edges^33^. These particles can significantly lower the induction time. The influence of contamination on the induction crystallization time was recently experimentally investigated in^35^. In this paper, a detailed molecular analysis of the surface of different particles was carried out and correlation dependences for the time of crystallization occurrence were determined. The solution was in the supersaturation state all the time. When the surface of the nanoparticles was free of impurities, the particles did not reduce the time of crystallization beginning. In the presence of contaminations, the induction time decreased many times during repeated experiments. To identify the relation for the induction time for the droplet and the layer, further studies using different micron particles in the form of contaminants are required, which is not the subject of this article.

Another characteristic feature of the graphs in Fig. 1(d) is the nonlinear nature of all curves. The theoretical expression for quasi-isothermal crystallization relating the parameters *K* and *P* has the form (5)^27^,

5 $$K \sim {(P)}^{0.5},\,{l}_{s} \sim {(t)}^{0.5},\,(ln{l}_{s})/(0.5lnt)=c$$

where *l*_*s*_ is the average distance that the crystallization front passes for time *t*. In logarithmic coordinates, all crystallization curves should have the form of a straight line with inclination *c* to the abscissa axis. With crystallization time, the salt concentration and the height of the drop change slightly (about 5%). Therefore, the thermal constants and the heat transfer coefficient will also vary slightly. In addition, the temperature jump on the crystallization front is less than 1 °C (measured by the thermal imager). However, from Fig. 1(d) it follows that for almost all curves *dK*/*dP* ≠ const. For the droplet *dK*/*dP* > 0. For the solution layer *dK*/*dP* < 0. The absence of a constant can be explained by the change in the supersaturation *S* over time. A solution layer covers the entire surface of the heater. When the crystalline film covers most of the surface of the salt solution (Fig. 1(c)), then the boundary thermal conditions change. Reducing the evaporation area increases the temperature under the solid film and in the vicinity of the crystallization front. The concentration in the solution increases slightly due to a slow evaporation at high salt concentration. As a result, the thermodynamic state of the salt solution in the vicinity of the crystallization front is shifted towards a smaller supersaturation *S*. At the same time, *j*_*cr*_ drops and tends to zero when approaching the hot side wall. The droplet occupies only a small part of the heater area. The drop diameter is 6 mm, and the heater diameter is 70 mm. The growth of the crystalline film on the droplet surface (Fig. 1(b)) does not cause a marked increase in the surface temperature. Then, the supersaturation and the temperature *T*_*s*_ are quasi-constant over the crystallization time. The sharp increase in the derivative *dK*/*dP* over time is associated with the curvature of the droplet contact line and the curvature of the droplet surface. This work does not mean to study in detail the effect of droplet curvature.

Figure 1(e) shows photos of salt crystallohydrates CaCl_2_·2H_2_O in the crystallization front and for different time moments *t*. As can be seen from the figures, crystallohydrates have the form of dendrites. Over time, the crystalline form changes. Experiments on crystallization in a thin layer of CaCl_2_ solution were carried out for different initial concentrations of salt *C*_*m*0_.

**Figure 2:**
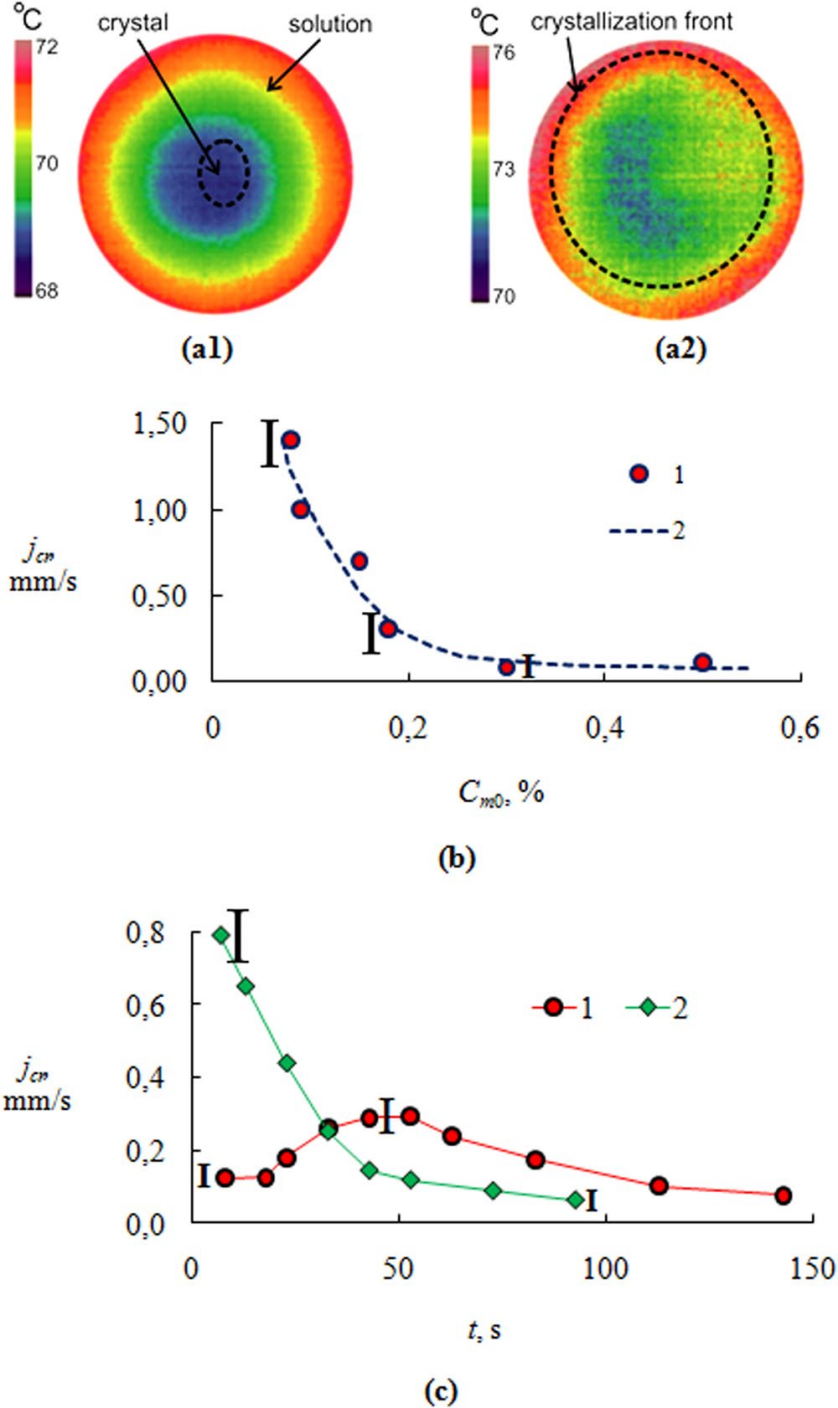
(**a**) Thermal images of the salt solution layer (**a1** – in the beginning of crystallization; **a2** – in the end of crystallization; (**b)** The layer crystallization rate *j*_*cr*_ depending on the initial mass concentration of CaCl_2_ salt *C*_*m*0_ (*T*_*w*_ = 75 °C); (**c)** The layer crystallization rate *j*_*cr*_ of CaCl_2_ salt solution over time (*T*_*w*_ = 75 °C): 1 – *C*_0_ = 35%; 2 – *C*_0_ = 13%.

Figure 2(a) shows the graph of change of *T*_*s*_ at crystallization on the free layer surface (thermal images for (**a1)** corresponds to the beginning of crystallization; (**a2)** corresponds the end of crystallization). The dotted line shows the boundary of the crystal film. When the crust covers most of the surface of the liquid, the temperature of the surface of the crust *T*_*s*_ increases by 2–4 °C.

Figure 2(b) presents an experimental dependence of *j*_*cr*_ on the initial salt concentration *C*_*m*0_. The crystallization rate *j*_*cr*_ = Δ*l*_*s*_/Δ*t* was determined by the slope of the straight line at the initial moment of the crystal front movement. The displacement Δ*l*_*s*_ was taken as the average displacement for the entire crystal front. Measurement of Δ*l*_*s*_ was carried out by processing the photos of the camera. In the photos, the phase boundary (liquid-crystallohydrate) was always clearly distinguishable. The curve *j*_*cr*_ = *f*(*C*_*m*0_) has a power form. For *C*_*m*0_ exceeding 30%, the crystallization rate slightly decreases. For *C*_*m*0_ values less than 20%, *j*_*cr*_ is very sensitive to changes in the initial salt concentration. Such a strong dependence of the crystallization kinetics on the concentration for low *C*_*m*0_ values is due to a higher evaporation rate. At the time of crystallization for low *C*_*m*0_ there was a small drop height *h* (less than 0.5 mm) and, accordingly, a higher heat flux from the wall. The higher *j*_*cr*_ value results in a higher degree of supersaturation during crystallization. For higher *C*_*m*0_ values, the induction time is weakly dependent on the initial salt concentration. As a result, supersaturation *S* and *j*_*cr*_ are quasi-constant.

Figure 2(c) shows the experimental curves of *j*_*cr*_ depending on time under different initial mass concentrations of CaCl_2_. Crystallization is realized on the free surface of the layer (Fig. 1(c)). For *C*_*m*0_ = 35%, the crystallization curve has an extremum. In the initial period of time the crystallization rate increases (*dS*/*dt* > 0), reaches a maximum and then falls. This behavior has been discussed above and is easily explained. When the crystal crust covers a negligible part of the liquid area (small times), the temperature *T*_*s*_ is quasi-constant. The concentration at the crystallization front increases with time, since the solution continues to evaporate and the salt concentration increases. However, with the growth of the crust area, the temperature *T*_*s*_ begins to rise markedly and the crystallization curve reaches the point at which *dS*/*dt* = 0 (the maximum point). With further crystallization, the growth of *T*_*s*_ (with a slight increase in salt concentration) leads to a noticeable drop in the supersaturation *dS*/*dt* < 0). When the crystallization front approaches the side wall of the heater, the crystallization curve tends to an extremum (minimum) when again *dS*/*dt* = 0.

Such a behavior with the presence of several extrema will always be observed in practice during the crystallization of solutions, if crystallization is accompanied by the following boundary conditions: (1) a thin film of a liquid solution or melt, (2) a high heat flux from the wall, (3) a relatively low crystallization rate and (4) a noticeable evaporation rate of volatile components.

Figure 2(c) also shows curve 2 for a relatively low value of *C*_*m*0_ = 13%. The high initial crystallization rate for *t* = 0 leads to a completely different behavior. The *j*_*cr*_ curve drops down sharply at 0 < *t* < 40 s and only when approaching the side wall of the heater the value of the derivative *dj*_*cr*_/*dt* approaches zero (*dS*/*dt* = 0). This behavior will be characteristic of solutions with a high initial crystallization rate and a low evaporation rate.

Thus, for the first group of solutions (curve 1) at the initial crystallization moment (*t* ≈ 0) the ratio *j*_*cr*_/*j*_*e*_ < 1 (*j*_*e*_ – evaporation rate) should be performed, and for the second group (curve 2) *j*_*cr*_/*j*_*e*_ > 1. When modeling crystallization, it is important not only to determine the value of the heat transfer coefficient, but also to establish the crystallization group, as well as to estimate the time change in the wall temperature *T*_*w*_ and the heat flux *q*_*w*_. The rates of change *dT*_*w*_/*dt* and *dq*_*w*_/*dt* will determine the character of crystallization. Often, technologies require amorphous structures or crystals with no long-range order. It is obvious that crystals which should exclude long-term dendritic growth on a free surface, need high values of a heat flux, high initial supersaturation (or overcooling), high density of crystal centers at homogeneous crystallization, as well as small thickness of a film of a solution. Such conditions are well implemented in the plasma spraying of nanofilms. High crystallization rates can lead to a strong heating of the solution under the crystalline film and to a sharp decrease in supersaturation due to the thermal inertia of the wall. Therefore, the oxidation of the wall or the formation of a wall layer of crystalline hydrates can significantly reduce the rate of crystallization due to a decrease in thermal conductivity and heat flux.

**Figure 3:**
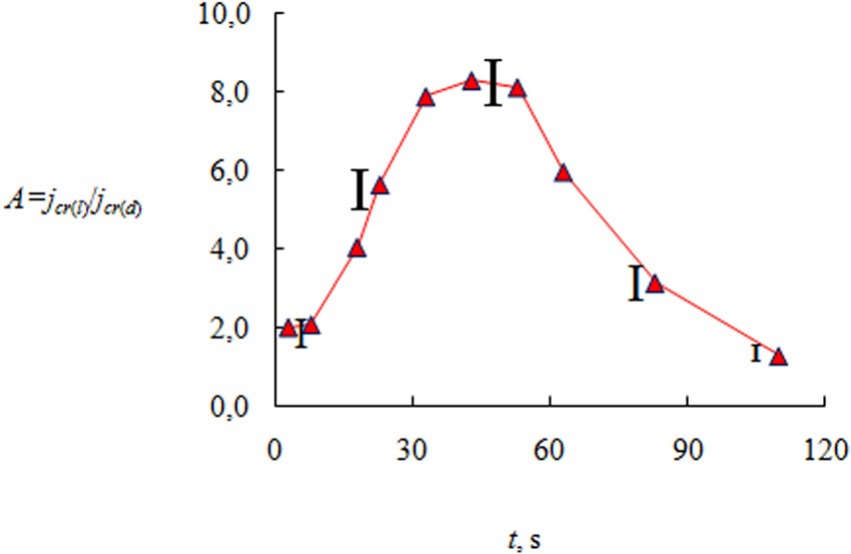
*A* = *jc*_*r*(*l*)_/*j*_*cr*(*d*)_ (initial height of a layer and a droplet during the crystallization *h*_0_ = 2.8 mm; *T*_*w*_ = 75 °C; *C*_*m*0_ = 35%, *j*_*cr*(*l*)_ is the crystallization rate for a layer (*l*) of salt solution, and *j*_*cr*(*d*)_ is the crystallization rate for the salt solution droplet (*d*).

Figure 1(d) demonstrates a comparison of the crystallization rate for a droplet and a layer in dimensionless coordinates. More clearly, this comparison may be presented in the form of the ratio *j*_*cr*(*l*)_/*j*_*cr*(*d*)_ (Fig. 3), where *j*_*cr*(*l*)_ is the crystallization rate for a thin layer of CaCl_2_ solution, and *j*_*cr*(*d*)_ is the crystallization rate for a drop of salt solution. As shown in Fig. 3, the parameter *A* = *j*_*cr*(*l*)_/*j*_*cr*(*d*)_ has an extremum (maximum). For the time interval 0 < *t* < 40–50 s, the curve increases (*dA*/*dt* > 0) and reaches maximum (*dA*/*dt* = 0). Next for *t* > 50–60 s, *dA*/*dt* < 0. For time *t* = 110–115 s, the parameter *A* becomes approximately equal to one (*j*_*cr*(*l*)_ ≈ *j*_*cr*(*d*)_). The maximum difference in the crystallization rates between the drop and the layer is approximately equal to 8. Such a non-linear behavior of the curve in Fig. 3 has been explained above and is due to different supersaturation for the droplet and layer, as well as to a different nature of *S* and *T*_*w*_ changes over time.

Let us consider the kinetics of growth of salt crystallohydrates in different directions on the surface of the droplet. Earlier it was indicated that the first active fast-growing crystallization centers occur near the contact line of the droplet. In the precursor film, the salt concentration and supersaturation are noticeably higher than at an insignificant distance from the contact line.

The crystallization process can be divided into several characteristic modes:

1. The growth of crystals, whose size becomes higher than the critical, in the precursor film. In the solution (before crystallization begins) there are much more crystalline nano-micro nuclei that have not reached a critical size. Spontaneous rapid growth is realized for crystals whose size has reached more than a critical value (Gibbs free energy reaches values that promote rapid growth). Thus, it can be reasonably assumed that the size of other crystals is several orders of magnitude smaller. The crystal growth time for a size of 0.1–1 mm is about 1 second. The growth of crystals along the contact line is one hundred seconds (order of values). Thus, the resulting visualization is quite enough.
2. When the crystal size becomes commensurate with the width of the precursor film, the growth of the crystal in the direction of the center of the droplet stops (or slows down many times). The crystal enters the region with *S* ≈ 1. The growth is realized in the direction (*l*_*cl*_) of the contact line (Fig. 4(a,b)), where the supersaturation is noticeably higher than *S* = 1. Time *t* = 0 s (Fig. 4(b,e)) corresponds to the beginning of crystallization. At *t* = 7 s, two crystals grow near the contact line in the *l*_*cl*_ direction (Fig. 4(b)). At *t* = 26 s, the crystals merge. A new crystal appears at a distance from the growing crystals at *t* = 30 s.

**Figure 4:**
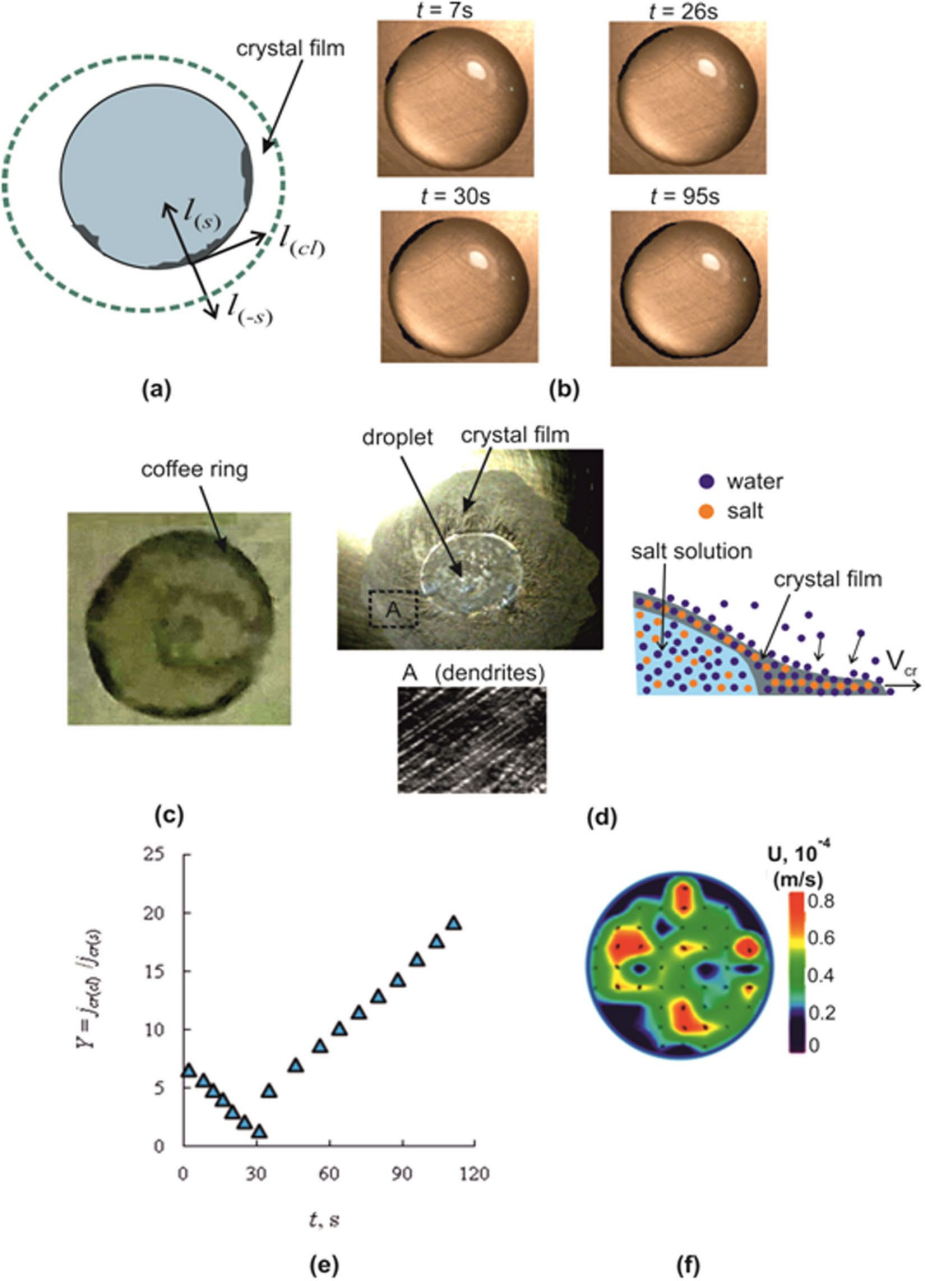
(**a**) Directions of crystallization; (**b**) Crystallization near the contact line of the droplet; (**c)** Coffee ring at the edges of the droplet; (**d)** Crystallization outside the droplet area; (**e**) The ratio of crystallization rates *j*_*cr*(*cl*)_/*j*_*cr*(*s*)_ in the direction of the drop contact line (cl) and in the direction of the center of the drop (s); (**f)** The Particle Image Velocity (PIV) measurements.
3. After the entire precursor film is covered with salt crystallohydrates, the growth of the film is realized in the direction (*l*_*s*_) of the drop center (Fig. 4(a)). The supersaturation *S* at the boundary of the crystallization front is still slightly above one). As a result of water evaporation from the surface of the droplet, the salt concentration and supersaturation increase and, accordingly, the crystallization rate increases with time. Due to the contact line curvature and the curvature of the droplet surface, the crystallization rate increases many times.
4. After the entire surface of the droplet is covered with a crust, the crystal film grows in the direction (*l*_***−s***_) (Fig. 4(d)) (the crystal front is shown in Fig. 4(a) with a dashed line).

At complete droplet evaporation in the contact line area, a “coffee spot” (deposition of micron-sized solid particulates) is formed due to the Marangoni flow^36^. In the case of evaporation of a drop of salt a coffee spot is difficult to identify due to the formation of crystals. For fixing the deposition a separate experiment was carried out. Before crystallization, a drop of salt solution was removed from the surface of the hot wall using a dispenser. Part of the remaining liquid was removed with cotton wool. At the edges of the drop the deposit was discernible (“coffee spot” in the region of the contact line of the drop (Fig. 4(c)). The formation of a deposit in the form of a ring at the edges of a drop for a salt solution, as well as for pure water, is associated with the circulation of the solution inside the drop due to the influence of the solutal and the thermal Marangoni flow.

In fact, the direction of growth of crystallohydrates in Fig. 4(b) occurs not only along the contact line of the droplet. The magnified image of the microscope shows that dendrites also grow slowly in the precursor film in the direction *l*_**(−s)**_ (Fig. 4(a)), opposite to the vector *l*_**(s)**_ (dendrites are removed from the center of the drop). If crystallization lasts for more than an hour, the area of the crystal film will increase 3–4 times. Figure 4(d) shows a photograph of the droplet surface and the resulting crystalline film outside the droplet area. The growth of the film is not obvious and requires physical explanation. Let us consider three possible mechanisms of crystal growth. Figure 4(d) schematically shows the growth of the crystal film on the dry surface of the metal wall. In the solution of the drop, the water concentration is higher than in salt crystallohydrates and under the action of diffusion, the water molecules can move in the direction of the edge of the film. The movement of water molecules is due to defects (cracks) inside the crystallohydrates (1 hypothesis), as well as at the movement of the molecular layer of water on the surface of the film (the second hypothesis). The third option may be associated with humid air. When drying the film of crystallohydrates, the bonds between the molecules of salt and water are broken and the crystallohydrate of the salt CaCl_2_·H2O breaks up. With further heating of the film, the thermodynamic equilibrium is disturbed and the evaporation of water is replaced by adsorption of water vapor. The destruction of the crystallohydrates allows the salt molecules to diffuse towards the edges of the film. It is characteristic that the crystallization rate *j*_cr(**−**s)_ in the direction *l*_(**−**s)_ is much lower than towards *l*_(s)_ (*j*_cr(s)_/*j*_cr(**−**s)_ ~ 0.01–0.02).

It should be also noted that the growth of dendrites in the direction of the center of the drop (*l*_(s)_) is not always realized. If crystallization in the droplet occurs on the wall without heating (*T* = 20–25 °C) and at high air humidity, the evaporation of the droplet stops (the salt solution reaches equilibrium on the free surface). The absence of evaporation will lead to the absence of supersaturation and stop the growth of dendrites in the direction of *l*_(s)_. In this paper, supersaturation in the vicinity of the droplet center increases due to high heat flux and relatively high evaporation rate.

The ratio of crystallization rates in two directions *l*_*cl*_ and *l*_*s*_ is presented in Fig. 4(e)). The rate of crystallization *j*_*cr*(*cl*)_ is taken along the contact line of the drop, and the rate *j*_*cr*(*s*)_ – in the direction of the center of the drop (*j*_*cr*(*cl*)_ = Δ*l*_*cl*_/Δ*t*, *j*_*cr*(*s*)_ = Δ*l*_*s*_/Δ*t*). Moreover, *j*_*cr*(*s*)_ is the average value of the rate for the full crystallization time (*j*_*cr*(*s*)_ = const). The *l*_*cl*_ displacement is taken as the total displacement from all growing centers. For time 0 < *t* < 26 s, the ratio *Y* decreases as the concentration and *S* between adjacent growing centers decreases. At the moment of appearance of the third center there is a jump in the rate *j*_*cr*(*cl*)_ and *j*_*cr*(*cl*)_ further increases due to the increase in *S*.

A significant increase in the salt concentration for *t* > 30 s is due to the extremely small height *h*_1_ of the solution in the precursor film (0.01–0.1 mm). As a result, convection in the film is suppressed. The free convention velocity in the precursor film has an order of *v*_*c*_ = 0.0001–0.001 mm/s (Micro PIV measurements). The diffusion rate can be estimated as a ratio of the diffusion coefficient to the layer height *v*_*d*_ = *D*/*h*_1_ = 0.0001 mm/s. Near the crystallization front (between neighboring growing centers), the salt concentration will fall. During crystallization, the salt concentration between closely spaced crystals decreases and this decrease does not have time to be compensated by diffusion. Away from the crystalline centers, the evaporation of water leads to the fact that the rate of evaporation (*v*_*e*_ = 0.01 mm/s) exceeds the rate of convection (*v*_*c*_ = 0.001 mm) (*v*_*ev*_ > *v*_*c*_). The slower the crystal front moves along the contact line, the greater the supersaturation at a distance from the front is.

During crystallization in the direction of the droplet center (*l*_*s*_), the solution height increases many times in comparison with the height of the precursor film. As a result, the role of convection increases many times and supersaturation in *l*_*s*_ direction will be lower than in the vicinity of the contact line. Measurements using the PIV method show that the average solution velocity in the horizontal section of the droplet has the order of 0.03–0.04 mm/s (Fig. 4(f)). A relatively high convective velocity in the bulk of the droplet will reduce the concentration gradient near the droplet interface. The increased concentration *C*_*s*_ will decrease due to the fact that near the wall the salt concentration is less. Therefore, the ratio *j*_*cr*(*cl*)_/*j*_*cr*(*s*)_ ≫ 1 will be realized. The maximum value of the *Y* parameter reflecting the crystallization anisotropy is approximately 20.

Thus, an important conclusion may be formulated from the experimental data on crystallization in a drop of salt solution. In the vicinity of the precursor film of a drop, a higher supersaturation *S* is set to due to the suppression of natural convention. As a result, the rate of crystallization in the direction of the contact line will be many times greater than the rate in the direction of the droplet center. In the bulk of the droplet, the convection velocity will be 30–50 times higher than at the edges of the droplet. Similar behavior and anisotropy will be also observed in other problems, where there is a triple phase boundary (liquid-solid-gas): the solution in the capillaries; the rod submerged in the liquid; the film of solution in contact with the side walls; a particle floating on a liquid surface, etc. These problems are frequent in modern technologies associated with crystallization and evaporation. Taking into account anisotropy will ensure a more correct modeling of the crystallization rate and the control over the properties of the obtained materials.

## Conclusion

The kinetics of crystallization in the drop is found to be fundamentally different from the thin layer (film). The appearance of the first growing crystal in the drop occurs near the contact line. In the salt solution film, the beginning of crystallization takes place near the center of the layer. The crystallization rate in the layer is many times higher than the velocity of the crystal front in the drop.

Experimental data show that the dependence of the crystallization rate *j*_*cr*_ on the initial mass concentration of salt *C*_*m*0_ is highly nonlinear. At low initial salt concentrations (*C*_*m*0_ < 20%), *j*_*cr*_ is highly sensitive to changes in *C*_*m*0_. For *C*_*m*0_ > 30%, *j*_*cr*_ is quasi-constant. This nonlinear character is determined by the supersaturation behavior in the solution.

For different experimental conditions, the heat transfer coefficients are determined to differ several times. Crystallization curves in dimensionless coordinates have been obtained taking into account the thermophysical properties of the solution and convection inside the liquid. A significant divergence of curves shows that it is not enough to take into account only the thermophysical properties and the heat transfer coefficient. The discrepancy between the experimental data is associated with a change in supersaturation *S* for the droplet and the layer, as well as with the dependence of the supersaturation on the initial salt concentration.

At the crystallization front there is a growth of dendrites both for drops, and for a layer. The form of dendrites changes over the crystallization time.

Anisotropy of crystallization is observed for a drop of salt solution. The rate of growth of salt crystallohydrate in the direction of the contact line is many times higher than the rate in the direction of the center of the droplet.

Four characteristic modes of crystallization to a drop of salt solution, which differ in the direction of dendrites motion and the rate of crystal growth, have been distinguished (for the second mode *j*_*cr*(*cl*)_/*j*_*cr*(−*s*)_ ≫ 1, for the third mode *j*_*cr*(*cl*)_/*j*_*cr*(*s*)_ ≫ 1, the fourth mode is characterized *j*_*cr*(*s*)_/*j*_*cr*(−*s*)_ ≫ 1).

The maximum ratio of the crystallization rate in the direction of the contact line to the rate in the direction of the droplet center reaches 20 times.

## References

[1] Nakoryakov, V. E. & Grigoryeva, N. I. Nonisothermal absorption in thermotransformers: Novosibirsk, Nauka (2010).

[2] Nakoryakov VE, Grigoryeva NI, Bartashevich MV (2011). Heat and mass transfer in the entrance region of the falling film: Absorption, desorption, condensation and evaporation. Int. J. Heat Mass Transf..

[3] Misyura SY (2016). Efficiency of methane hydrate combustion for different types of oxidizer flow. Energy.

[4] Kuznetsov GV, Feoktistov DV, Orlova EG (2016). Regimes of spreading of a water droplet over substrates with varying wettability. J. Eng. Phys. Thermophy..

[5] Wang Y, Wang Z-G (2018). Droplets wetting and evaporating on ethanol-philic micro-structured surfaces. Int. J. Heat Mass Transf..

[6] Gao M (2017). An experimental investigation of sessile droplets evaporation on hydrophilic and hydrophobic heating surface with constant heat flux. Int. Commun. Heat Mass Transf..

[7] Schmid J (2018). Crystallization of urea from an evaporative aqueous solution sessile droplet at sub-boiling temperatures and surfaces with different wettability. Exp. Therm. Fluid Sci..

[8] Wagner R (1997). Siliconmodified carbohydrate surfactants: IV. The impact of substructures on the wetting behavior of siloxanyl-modified carbohydrate surfactants on low-energy surfaces. Appl. Organomet. Chem..

[9] Semenov S (2013). Evaporation of droplets of surfactant solutions. Langmuir.

[10] Ibarra-Bahena J (2017). Experimental assessment of a hydrophobic membrane-based desorber/condenser with H_2_O/LiBr mixture for absorption systems. Exp. Therm. Fluid Sci..

[11] Camassel B (2005). Evaporation in capillary tube of square cross-section: application to ion transport. Chem. Eng. Sci..

[12] Zheng Z, Song S, Wang Y (2016). Sol-gel-processed amorphous lithium ion electrolyte thin films: Structural evolution, theoretical considerations, and ion transport processes. Solid State Ionics.

[13] Misyura SY (2018). Non-isothermal evaporation in a sessile droplet of water-salt solution. Intern. J. Therm. Sci..

[14] Misyura SY (2015). High temperature nonisothermal desorption in a water-salt droplet. Int. J. Therm. Sci..

[15] Misyura SY (2017). Evaporation of a sessile water drop and a drop of aqueous salt solution. Sci. Rep..

[16] Nakoryakov VE (2014). Nucleate boiling in pure-water and salt-water droplets. Doklady Physics.

[17] Conde MR (2004). Properties of aqueous solution of lithium and calcium chlorides: formulations for use in air conditioning equipment design. Int. J. Therm. Sci..

[18] Sudzuki, K., Fudzimori, H. & Khasimoto, K. Amorphous metals: Moscow, Metallurgia (1987).

[19] Kunitskiy, Y. A., Korzhik, V. N. & Borisov, Y. S. Non-crystalline metal materials and coatings in engineering: Kiev, Tekhnika (1988).

[20] Misyura SY (2018). The anomalously high rate of crystallization, controlled by crystal forms under the conditions of a limited liquid volume. Cryst. Growth Des..

[21] Qu G, Kwok JJ, Diao Y (2016). Flow-directed crystallization for printed electronics. Acc. Chem. Res..

[22] Shahidzadeh-Bonn N (2008). Salt crystallization during evaporation: impact of interfacial properties. Langmuir.

[23] Linnow K (2013). *In situ* Raman observation of the crystallization in NaNO_3_-Na_2_SO_4_-H_2_O solution droplet. Environ. Earth Sci..

[24] Shahidzadeh N (2015). Salt stains from evaporating droplets. Sci. Rep..

[25] Kuznetsov GV (2018). Evaporation modes of LiBr, CaCl_2_, LiCl, NaCl aqueous salt solution droplets on aluminum surface. Int. J. Heat Mass Transf..

[26] Semenov ME (2015). DSC and thermal imaging studies of methane hydrate formation and dissociation in water emulsions in crude oils. J. Therm. Anal. Calorim..

[27] Mullin, J.W. Crystallization: 4^th^ ed. Butterworth, Oxford (2004).

[28] Kelly-Zion PL (2011). Evaporation of sessile drops under combined diffusion and natural convection. Colloid Surf. A.

[29] Misyura SY (2018). Evaporation and heat transfer of aqueous salt solutions during crystallization. Appl. Them. Eng..

[30] Carle F (2016). Contribution of convective transport to evaporation of sessile droplets: Empirical model. Int. J. of Therm. Sci..

[31] Strizhak PA (2018). The role of convection in gas and liquid phases at droplet evaporation. Int. J. of Therm. Sci..

[32] Volkov RS (2018). The influence of key factors on the heat and mass transfer of a sessile droplet. Exp. Therm. Fluid Sci..

[33] Hu H, Larson RG (2006). Marangoni effect reverses coffee-ring depositions. J. Phys. Chem. B..

[34] Lyubov, B. Y. Theory of crystallization in large volumes: Moscow, Nauka Publishers (1975).

[35] Nesterov AN (2015). Promotion and inhibition of gas hydrate formation by oxide powders. J. of Molecular Liquids.

[36] Deegan RD (1997). Capillary flow as the cause of ring stains from dried liquid drops. Nature.

